# Bone Fusion in the Cervical Spine: Where Are We Now?

**DOI:** 10.3390/bioengineering13060614

**Published:** 2026-05-25

**Authors:** Maria Caterina Evangelisti, Alida Mazzoli, Ivan Cabrilo, Giuseppe Perale

**Affiliations:** 1 Department of Science and Engineering of Matter, Environment and Urban Planning (SIMAU), Università Politecnica delle Marche, UdR INSTM, Via Brecce Bianche, 60131 Ancona, Italy; a.mazzoli@staff.univpm.it; 2 Department of Neurosurgery, Neurocenter of Southern Switzerland, Ente Ospedaliero Cantonale, 6900 Lugano, Switzerland; ivan.cabrilo@eoc.ch; 3 Industrie Biomediche Insubri SA, Via Cantonale 67, 6805 Mezzovico-Vira, Switzerland; giuseppe@ibi-sa.com; 4 Faculty of Biomedical Sciences, University of Southern Switzerland (USI), Via G. Buffi 13, 6900 Lugano, Switzerland; 5 Ludwig Boltzmann Institute for Experimental and Clinical Traumatology, Donaueschingenstrasse 13, 1200 Vienna, Austria

**Keywords:** bone fusion, cervical spine, neurosurgery, bone graft, artificial intelligence, imaging analysis

## Abstract

Anterior cervical discectomy and fusion (ACDF) is one of the most commonly performed surgical procedures for the treatment of cervical degenerative disease, myelopathy, radiculopathy, and segmental instability. Although clinical outcomes are generally favorable, pseudarthrosis remains a relevant complication, with a reported incidence ranging from 5% to 20%. In a field with no yet clear main directions, this narrative review aims at giving the reader a broad picture and a wide analysis of the recent advances in cervical spinal fusion, with particular focus on biomaterials, intervertebral cage technologies, cervical spine biomechanics and imaging methods used for fusion assessment. The literature regarding quantitative imaging parameters and emerging applications of artificial intelligence (AI) is also reviewed. Current bone grafts include autologous grafts, allografts, xenografts and polymeric grafts, while the materials for the intervertebral cages comprehend titanium, polyetheretherketone and silicon nitride, with reported fusion rates distributed in a very large range. Computed tomography (CT) remains the standard imaging modality to assess whether fusion has occurred, due to its high spatial resolution. However, the lack of shared diagnostic criteria and the significant interobserver variability continue to limit its reliability. Quantitative parameters, such as Hounsfield Unit measurements and MRI-derived bone quality scores, may contribute to a more objective evaluation, although current evidence remains heterogeneous. In parallel, AI-based imaging analysis is showing promising results for quantitative assessment and longitudinal monitoring of bone fusion; however, large prospective clinical studies are still needed to confirm its clinical applicability. In conclusion, despite advances in surgical technologies and biomaterials, radiological assessment of cervical fusion still lacks universally accepted diagnostic standards. Future AI applications may improve diagnostic accuracy and reproducibility, promoting a more standardized approach in clinical practice.

## 1. Introduction

Bone fusion is a surgical process in which motion between two bones is eliminated by promoting their biological union, resulting in a single, rigid structure. In the spine, fusion between adjacent vertebrae prevents intervertebral movement at a specific level and is typically indicated in patients experiencing pain or other symptoms associated with pathological motion [1].

Cervical spine fusion is widely adopted in several clinical scenarios. For example, it is often performed in the context of anterior decompressive procedures in which discoligamentous and bony structures are removed to relieve pressure off neural elements, thereby requiring stabilization. This procedure is referred to as anterior cervical discectomy and fusion (ACDF) and is one of the most frequent procedures for the treatment of cervical spine disorders and alleviating symptoms such as pain, numbness, weakness, and paresthesia in the upper limbs [2,3,4].

Indications for ACDF include herniated or degenerated discs causing nerve root compression or cervical myelopathy, which can manifest with arm and neck pain, neurological deficits such as numbness or weakness, balance or gait disturbances, and sphincter incontinence [4].

The procedure is performed under general anesthesia, through an anterior cervical approach and takes approximately one hour, although this may vary depending on surgical complexity and number of spinal segments. It involves the removal of the damaged disc and preparation of the intervertebral space. Subsequently, a bone graft—autologous, allogenic, or synthetic—or a cage filled with graft material is inserted between the vertebrae. Additional stabilization may be achieved using anterior plating with screws or cage-integrated fixation systems. Postoperatively, over time new bone gradually forms through and around the graft, resulting in permanent fusion of the vertebrae into a single solid unit and elimination of motion at that level.

Spinal fusion is therefore a permanent and irreversible procedure. The risk of postoperative complications, including secondary bleeding or infection, is relatively low, and neurological complications are uncommon [2].

The primary objective of spinal fusion is to achieve a stable spinal segment by eliminating pathological motion while preserving acceptable mobility of adjacent segments. Reported clinical and radiological success rates are high in the cervical spine, with most patients experiencing significant pain reduction and decreased reliance on analgesic medications following surgery [2,3]. While clinical success is evaluated in terms of improvement of the initial clinical presentation, radiological success is defined by the achievement of fusion. These outcomes do not necessarily always correlate; patients without complete fusion may still experience clinical improvement, whereas others with confirmed fusion may remain symptomatic. The latter scenario may actually call into question the underlying origin of the patient’s pain. Nevertheless, achieving fusion is generally considered a marker of procedural success, as it effectively excludes pseudarthrosis from the differential diagnosis of postoperative pain.

Several factors may negatively influence fusion success, including the anatomical location and number of fused segments [5,6,7], local vascular supply, and pharmacological treatments. For example, nonsteroidal anti-inflammatory drugs (NSAIDs) have been shown to impair osteogenesis and bone healing [5,8]. The results of an investigation conducted by Martin et al. [8] indicate that the administration of a commonly prescribed nonsteroidal anti-inflammatory drug negatively influences spinal fusion during the early postoperative period, as demonstrated in a well-established rabbit model of posterolateral lumbar fusion. Notably, the use of recombinant bone morphogenetic protein-2 in combination with autologous bone graft was able to mitigate the suppressive effect of ketorolac on new bone formation.

The main concern regarding the use of NSAIDs in bone healing stems from their inhibitory effect on prostaglandin synthesis; however, a clear molecular mechanism underlying this relationship has not yet been fully established [9]. Furthermore, a recent retrospective study found that the use of NSAIDs and COX-2 inhibitors in the early postoperative period is associated with a clinically significant increase in rates of pseudarthrosis, hardware failure, and the need for revision surgery in patients undergoing non-segmented posterior spinal instrumentation and fusion [10].

In light of these findings, the avoidance of long-term nonsteroidal anti-inflammatory drugs for postoperative analgesia in patients undergoing spinal arthrodesis may play a role in their postoperative management [8,9,10].

Currently, the degree of postoperative spinal fusion is assessed using advanced radiological imaging techniques [11]. Computed tomography (CT) is widely used for evaluating bone fusion, as it allows detailed visualization of osseous bridging between adjacent vertebrae, trabecular continuity across the fusion site, absence of radiolucent gaps, and integration of the graft or intervertebral cage.

Dynamic radiographs are also routinely used during follow-up to assess segmental stability, particularly by evaluating the absence of abnormal motion between vertebrae during flexion–extension movements.

Brantigan et al. [12] demonstrated a strong correlation between post-mortem radiographs, CT findings, and histological analysis when radiolucent cages were used. However, a major limitation in the assessment of spinal fusion is the lack of standardized diagnostic criteria. Existing studies adopt heterogeneous evaluation methods and thresholds, resulting in significant interobserver variability [11,13], which complicates objective determination of fusion status [5,14].

On conventional radiographs, the presence of a bone bridge at the graft–vertebral interface may be observed; however, this finding shows limited reproducibility and reliability [11]. Moreover, evaluation of fusion involving posterior spinal elements remains particularly challenging using standard radiographic projections due to the small size and complex anatomy of these structures [13,15].

Failure of bone fusion is defined as pseudarthrosis. Among preventive strategies, the use of vascularized bone grafts has been shown to enhance biological integration and improve healing outcomes.

In the cervical spine, achieving stable fusion at the craniocervical junction remains particularly challenging due to the high mobility of the occiput–C1 and C1–C2 joints, as well as the limited availability of posterior bony surfaces suitable for decortication and graft placement [15,16,17,18].

More recently, artificial intelligence (AI) has emerged as a promising tool for the automated analysis of spinal imaging, including MRI, CT, and PET scans, through deep learning-based models.

This study was designed as a structured narrative review, incorporating methodological elements derived from systematic reviews to improve transparency, reproducibility, and rigor. A comprehensive literature search was performed across multiple electronic databases, including PubMed, Scopus, Web of Science, EMBASE, and CINAHL Plus, covering publications up to January 2026.

The search strategy combined Medical Subject Headings (MeSH) and free-text terms related to cervical spinal fusion and associated technologies. The following keywords were used in different combinations with Boolean operators (AND/OR): “cervical spinal fusion”, “bone graft”, “spine surgery”, “artificial intelligence”, and “spine radiology.”

Additional relevant studies were identified through manual screening of reference lists of the included articles.

A formal risk of bias assessment was not performed due to the heterogeneity of the included studies and the predominance of retrospective and non-randomized designs.

However, potential sources of bias were considered, including variability in fusion assessment criteria, differences in imaging protocols, heterogeneity in follow-up duration, and inconsistencies in outcome reporting. These limitations were taken into account in the interpretation of the findings.

The selected studies included meta-analyses, systematic reviews, narrative reviews, and clinical studies. Preclinical studies, including in vitro and animal models, were included. Only studies involving patients undergoing cervical spinal fusion were considered.

The aim of this review is to provide an updated overview of cervical spinal fusion, focusing on recent advances in biomaterials. The second key aspect is to highlight a critical limitation in current clinical practice: the absence of standardized and reliable imaging-based criteria for evaluating fusion status. For this reason, a review of novel AI systems developed in recent years to improve the analysis of radiological images is presented, noting that the literature in this field remains highly inconsistent.

## 2. Bone Grafts and Intervertebral Cages

Bone grafts currently used in clinical practice can be broadly classified into three main categories: biological grafts, naturally derived materials, and synthetic grafts. Biological grafts include autografts, allografts, and xenografts, which are obtained from living or previously living tissues. In contrast, naturally derived and synthetic grafts are produced from materials of natural origin (e.g., collagen, gelatin, alginate) or fully synthetic polymers (e.g., polycaprolactone, polylactic acid, and polymethylmethacrylate). In addition, hybrid grafts have been developed by combining biological and synthetic components to enhance both mechanical and biological performance.

Although numerous biomaterials and implant technologies have been proposed for ACDF, robust cervical spine-specific comparative evidence remains limited and does not allow a critical comparative analysis. This work provides an overview of the main bone graft substitutes currently used in general orthopedic practice and reviews the evidence when applied to ACDF. Various commercial products already described in the literature are also presented, in order to provide a comprehensive appraisal of commercially available materials and implants.

### 2.1. Autologous Graft

Autologous grafts, particularly cancellous bone, remain among the most widely used options and have demonstrated high efficacy in the treatment of both septic and aseptic non-unions, as well as long-bone fractures [19,20,21].

However, cancellous grafts provide limited mechanical support, as they undergo substantial resorption within approximately one year, despite their rapid revascularization and excellent biological integration.

In contrast, cortical grafts offer superior structural stability, although their remodeling and replacement by new bone occur more slowly due to their dense structure. Vascularized cortical grafts, although technically more demanding to harvest and implant, provide faster healing and improved cellular viability compared with non-vascularized grafts [19,22]. During the first six weeks following implantation, vascularized grafts exhibit greater mechanical strength and biological activity than non-vascularized grafts.

The success of autografts largely depends on the preservation of osteoprogenitor cells and viable osteocytes; excessive manipulation or delayed implantation may compromise graft viability and clinical outcomes [19,23].

In anterior cervical discectomy and fusion (ACDF) procedures, the autologous iliac crest bone graft (ICBG) has historically been considered the gold standard due to its osteogenic, osteoinductive, and osteoconductive properties.

### 2.2. Allograft

Allografts are derived from living donors or cadaveric bone and represent a widely accepted alternative to autografts. They are typically available in three forms: fresh, fresh-frozen, and freeze-dried [20].

Allografts can be further classified into cancellous, cortical, and demineralized bone matrix (DBM) types [19]. Unlike autografts, cancellous allografts primarily provide osteoconductive support due to the absence of viable osteogenic cells.

Compared with autografts, allografts are associated with higher failure rates, mainly due to immunogenicity and the risk of host immune rejection mediated by major histocompatibility complex (MHC) antigens. Additional limitations include reduced osteoinductive capacity, potential cytotoxic effects related to sterilization processes, and the risk of disease transmission [22]. Moraschini et al. reported that approximately 48% of patients remained immunologically sensitized following allogenic bone grafting [19].

Demineralized bone matrix (DBM) is a specific type of allograft in which the mineral phase is removed while preserving the organic matrix and associated growth factors. However, DBM presents several limitations, including variability in osteoinductive potential and potential loss of biological activity during processing and sterilization [24]. Furthermore, reduced structural integrity may lead to particle migration, potentially interfering with vascularization and graft integration [19,25]. For this reason, it cannot support the disc space on its own and so it requires being used with mineralized allograft or synthetic cage [3].

Currently, no DBM product fully meets the criteria of an ideal bone graft substitute, and significant variability exists among commercially available products, even when similar processing methods are employed [3,19,26].

In multilevel ACDF procedures, allograft cellular bone matrices (ACBMs) have demonstrated promising outcomes and may represent a viable alternative to autografts, particularly in patients with compromised bone quality [27]. Indeed, in a study conducted by Goldman et al. involving a total of 73 patients, 45% of those with available imaging showed complete fusion at 6 months, whereas this proportion increased to 97.4% at 12 months among patients with available imaging [27].

Commonly used commercial allografts include: Bio4^®^, Osteofil^®^ DBM, Grafton^®^ DBM and DBX^®^ DBM Putty. For completeness, the authors report a comparative study, although it is focused on posterolateral lumbar fusion, evaluating Grafton^®^ DBM and DBX^®^ DBM: it reported fusion rates of approximately ~70.4% (Grafton) vs. ~66.7% (DBX) respectively at 24-month follow-up [28]. Overall, despite the widespread use of allografts in ACDF, the current literature remains heterogeneous, with limited prospective comparative studies specifically focused on cervical fusion outcomes.

### 2.3. Xenograft

Xenografts are derived from animal sources and primarily provide osteoconductive support. Their use eliminates the need for a secondary surgical site for autologous bone harvesting and does not significantly prolong surgical time or recovery [29].

Decellularized xenogeneic bone scaffolds retain a native-like microarchitecture, exhibiting mechanical properties, chemical composition, porosity, and pore size distribution comparable to human bone [19]. Among currently available options, decellularized cancellous xenografts represent a promising alternative to autografts due to their structural similarity to native bone tissue.

Additionally, their wide availability, adaptability to different geometries, and cost-effectiveness make xenografts attractive for large-scale production and clinical application [30].

Several xenogeneic graft substitutes are commercially available. For example, Kiel Surgibone^®^ has been used in ACDF procedures, demonstrating fusion rates comparable to autografts in multiple studies [31]. Other available products include CANCELLO-PURE^®^ and BIO-GEN^®^; however, robust clinical evidence specifically evaluating their performance in spinal fusion remains limited, particularly in multicenter clinical studies.

### 2.4. Natural Biopolymers Graft

In bone tissue engineering, a wide range of natural polymers has been investigated for regenerative applications, including collagen, sodium alginate, hyaluronic acid, silk fibroin, and chitosan.

Collagen is a major component of native bone and is widely used due to its excellent biocompatibility and bioactivity. It is available in various forms, including membranes, sponges, and hydrogels [32]. Collagen membranes are commonly employed in guided bone regeneration (GBR); however, their limited mechanical strength and rapid degradation represent significant drawbacks [33].

Gelatin-based biomaterials are also widely used, often in combination with other materials to enhance mechanical stability and biological performance [34].

These materials can be administered through minimally invasive injection techniques, enabling localized treatment while reducing surgical trauma. However, natural polymers present several limitations, particularly in controlling degradation rates. Rapid degradation, commonly observed in collagen- and gelatin-based scaffolds, may result in premature loss of mechanical integrity before sufficient bone formation occurs. Conversely, slower-degrading materials such as alginate may delay replacement by newly formed bone, potentially impairing integration and, in some cases, promoting chronic inflammatory responses [19].

### 2.5. Synthetic Graft

Synthetic bone grafts encompass a wide range of materials, including metals, bioceramics, bioglass, and synthetic polymers. These materials can be manufactured in various forms, such as moldable pastes, granules, injectable formulations, and three-dimensional (3D)-printed scaffolds [19,22].

Moldable and granular grafts are particularly advantageous in traumatic conditions or in clinical scenarios requiring immediate intervention, where sufficient time for the design and fabrication of patient-specific 3D-printed constructs may not be available [19,22].

Over the past four decades, calcium phosphate (CaP) ceramics have become widely used as bone substitutes in orthopedic surgery due to their excellent biocompatibility, osteoconductive properties, and chemical similarity to the mineral phase of native bone [35].

Advances in additive manufacturing have further enabled the production of patient-specific implants. Customized prostheses fabricated using trabecular titanium through 3D-printing technologies have been successfully applied in clinical practice, allowing improved anatomical reconstruction, implant fit, and primary stability [36].

However, bioceramic powders are generally unsuitable for direct clinical application due to their rapid degradation and associated volume loss, which may compromise structural stability. To address this limitation, porous three-dimensional scaffolds have been developed to promote bone ingrowth and enhance mechanical performance [37].

Hydroxyapatite (HAp) bioceramics are among the most widely used materials for bone substitution due to their close resemblance to the mineral phase of native bone. Although porous HAp implants exhibit relatively low initial mechanical strength, their mechanical properties improve significantly with progressive bone ingrowth. When approximately 50–60% of the pore volume is filled with cortical bone, bending strength can increase to approximately 40–60 MPa [38].

Despite these advantages, conventional HAp presents limitations, including low biodegradability and intrinsic brittleness, which may restrict long-term remodeling. To overcome these drawbacks, nanocrystalline HAp has been developed, as its increased surface-area-to-volume ratio enhances biological activity and resorption kinetics.

Several commercially available DBM and cellular bone matrices have been proposed for ACDF; however, comparative cervical spine-specific evidence remains limited and heterogeneous [39,40,41].

In addition, bioactive glass-based grafts have gained increasing attention in spinal fusion. For example, a promising option is represented by Signafuse^®^ [42]: although it has not yet been applied in ACDF cases according to the current literature, it has demonstrated excellent results in lumbar fusion. In fact, Seaman et al. reported a fusion rate of 87.5% in patients treated with Signafuse^®^.

Among synthetic polymers, polyglycolic acid (PGA) is widely used due to its favorable mechanical properties, biodegradability, and biocompatibility [43]. PGA-based membranes have shown effectiveness in supporting bone regeneration in guided bone regeneration (GBR) procedures [44,45].

To improve mechanical performance and control degradation rates, PGA is often copolymerized with other polymers, such as polylactic acid (PLA), forming poly(lactic-co-glycolic acid) (PLGA), which is extensively used in tissue engineering applications [46].

Poly(methyl methacrylate) (PMMA) has also been extensively investigated for biomedical applications, including composite biomaterials and 3D-printed implants [47,48]. The incorporation of reinforcing agents, such as tantalum carbide (TaC), significantly enhances mechanical strength, radiopacity, and osteogenic potential without compromising biocompatibility [19]. In particular, TaC–PMMA composites have demonstrated compressive strengths exceeding 100 MPa and radiopacity comparable to clinically used bone graft substitutes [49].

### 2.6. Hybrid Graft

Hybrid or composite biomaterials are formed by combining two or more constituents with distinct physical and chemical properties. Typically, a continuous matrix phase provides the structural framework, while a dispersed reinforcing phase—such as particles or fibers—enhances mechanical strength and biological performance [19].

Compared with single-component materials, composite scaffolds generally exhibit improved functionality, offering greater control over biodegradability, osteoconductivity, and mechanical stability [50].

In recent years, the combination of synthetic materials with xenografts has emerged as a promising strategy with increasing clinical relevance. This approach enables the integration of the biological advantages of xenogeneic matrices—such as biocompatibility and osteoconductive potential—with the superior mechanical properties and tunable degradation profiles of synthetic components [19,51].

Synthetic components may also improve structural stability and allow better control of scaffold resorption kinetics while preserving the biological characteristics of the xenograft matrix.

Recent developments include the incorporation of resorbable polymers into xenogeneic scaffolds to further enhance their performance. Several hybrid graft substitutes are currently available, including SmartBone^®^ ORTHO and Hypro-Oss^®^ ORTHO [52,53,54].

However, high-quality controlled clinical studies specifically evaluating their performance in cervical spinal fusion remain limited. Similarly, Tutobone^®^ has been investigated as a bone graft substitute [55], although evidence in ACDF procedures remains scarce, with reported fusion rates generally lower than those achieved with autografts but comparable to other substitutes in terms of overall clinical outcomes [56].

### 2.7. Intervertebral Cages

Intervertebral cages are implantable spacer devices positioned between adjacent vertebrae following disc removal during ACDF procedures.

Bone graft material is placed within the cage to provide immediate mechanical stability while promoting bone fusion [57].

In recent years, ACDF procedures performed without cages have become increasingly uncommon. Cages may be used in combination with anterior plate fixation to enhance stability or with cage-integrated screw fixation systems; however, stand-alone cages have gained popularity, particularly in single-level procedures. In more complex or multilevel cases, cages are often supplemented with posterior fixation to ensure adequate biomechanical support and enhance fusion rates Indeed, the rate of non-fusion has been shown to increase with the number of operated segments [58]. The most used materials for cervical intervertebral cages include titanium and its alloys, polyetheretherketone (PEEK), and carbon fiber-reinforced polymers [59].

PEEK offers several advantages, including radiolucency, which facilitates postoperative imaging, and an elastic modulus similar to that of cortical bone, thereby reducing stress shielding. As a result, it has been widely adopted in clinical practice.

Titanium cages, particularly those with porous architectures or produced using additive manufacturing techniques, exhibit superior osseointegration due to their bioactive surface properties. However, their higher stiffness compared with bone may influence load distribution.

To combine the advantages of both materials, hybrid cage systems—such as titanium-coated PEEK implants—have been developed. These systems aim to improve osteointegration while maintaining favorable mechanical compatibility. For example, fusion rates of up to 94.1% have been reported in patients treated with titanium-coated PEEK cages in ACDF procedures [60].

Conversely, other studies have reported higher fusion rates with standard PEEK cages compared with titanium-coated variants at 12 months postoperatively, suggesting that surface modifications may influence biological response and clinical outcomes [60].

Furthermore, particularly in patients with reduced bone mineral density, lattice-structured cages designed to mimic the trabecular architecture of cancellous bone have demonstrated improved biomechanical performance and a reduced risk of postoperative subsidence [61].

In recent years, also silicon nitride has been used. It is a highly effective bioceramic material whose strength and toughness make it particularly suitable for implant applications [62,63]. It is considered an advantageous biomaterial due to its mechanical reliability, as well as its ability to promote osteoconductivity, exhibit bacteriostatic properties, and reduce the risk of cage subsidence in the cervical spine. Additionally, silicon nitride offers improved radiolucency and wear resistance, while facilitating successful spinal fusion between the implant and adjacent vertebrae [62,63]. Overall, this bioceramic contributes to both the structural stability and biological performance of the implant.

To improve the clinical interpretability of the available literature, the main graft materials and intervertebral cage technologies are summarized in Table 1.

## 3. Influence of Biomechanics on Cervical Fusion

The cervical spine is uniquely designed to support the weight of the head while allowing a high degree of mobility, making it biomechanically sensitive to alignment changes [64].

In cervical fusion, the elimination of motion at one or more segments leads to a redistribution of mechanical stresses to adjacent levels, which may accelerate degeneration or alter kinematics if alignment is not properly restored [65].

A key concept in this context is cervical sagittal balance, defined as the ability to maintain horizontal gaze with minimal muscular effort. It is primarily described by cervical lordosis (CL), sagittal vertical axis (SVA), and T1 slope (T1S), which reflects the inclination of the thoracic inlet and is closely related to the amount of lordosis required [66].

Loss of cervical balance, such as postoperative kyphosis or insufficient lordosis, increases muscular demand on the posterior elements and may result in pain and fatigue [67]. A strong correlation between T1 slope and cervical lordosis has been demonstrated; therefore, a mismatch between these parameters may indicate sagittal imbalance, reflecting a loss of harmonious cervical alignment and increased biomechanical demand to maintain horizontal gaze [68,69]. In the context of cervical fusion, inadequate restoration of sagittal alignment may lead to anterior translation of the head and increased SVA, thereby altering cervical biomechanics [65].

However, the relationship between postoperative cervical alignment and clinical outcomes remains controversial, as several studies have not found a significant correlation between sagittal parameters and patient-reported outcomes after ACDF [70].

Biomechanical considerations also influence implant selection and fixation strategies, which aim to ensure segmental stability while optimizing load sharing between the interbody device and the anterior column [71]. The mechanical properties of cervical constructs are dependent on cage geometry, material composition, and the use of supplemental anterior plating, all of which determine overall construct stiffness and stress distribution across the fusion interface. Excessive rigidity may result in stress shielding, thereby reducing physiological mechanical stimulation required for osteogenesis, whereas insufficient stability may allow excessive micromotion at the graft-endplate interface, exceeding the threshold compatible with bone formation and leading to fibrous union rather than solid arthrodesis [71]. Therefore, successful fusion relies on achieving an optimal biomechanical environment that balances stability with controlled mechanical loading.

The number of fused levels further affects global spinal biomechanics. Multilevel fusions are associated with greater alterations in sagittal alignment and may require compensatory mechanisms in adjacent segments or the thoracic spine [65]. Extension of fusion constructs into the thoracic spine is sometimes considered to better control sagittal balance, particularly in patients with high T1 slope [67].

Paraspinal muscles also play an important role in maintaining alignment. Degeneration or dysfunction of these structures may impair the ability to sustain cervical balance even after technically successful fusion [69].

Overall, restoration of cervical sagittal balance allows for more efficient load distribution, preservation of horizontal gaze, and improved clinical outcomes, whereas imbalance may result in persistent symptoms and long-term complications [72].

In summary, biomechanics play a central role in cervical fusion by regulating postoperative load transmission across spinal segments. While biomechanical considerations suggest that sagittal balance parameters and malalignment may in theory influence the mechanical environment at the fusion site, and thereby affect conditions for osteogenesis, clinical studies have not consistently demonstrated a clear association between cervical alignment parameters and radiological fusion outcomes as exists in the lumbar spine. Nevertheless, several studies do indeed indicate towards such an association [73,74]. For example, changes in the pre- and post-operative C2–C7 Cobb angle have been identified as predictors of fusion outcomes. Increased cervical lordosis may improve load distribution and enhance mechanical stability, thereby creating a biomechanical environment more favorable for bone formation and fusion [73,75].

## 4. Pseudarthrosis

Despite the ACDF procedure’s generally satisfactory outcomes [2,3], several postoperative complications may nevertheless occur.

Over time, fusion of adjacent vertebral segments may alter physiological load distribution, leading to increased mechanical stress and progressive degeneration of neighboring levels, a condition referred to as adjacent segment disease (ASD). The reported incidence of ASD varies widely, ranging from 0.4% to 32%, with cigarette smoking identified as one of the most significant risk factors [76]. To mitigate these effects, motion-preserving strategies, such as cervical disc arthroplasty, have been developed as alternatives to fusion, aiming to restore spinal function while reducing the risk of adjacent segment degeneration [77].

Pseudarthrosis remains one of the most significant complications following spinal fusion. It is defined as the failure to achieve solid osseous union between adjacent vertebrae and may result in persistent pain, mechanical instability, and, in severe cases, the need for revision surgery [78]. Clinical studies report pseudarthrosis depends on surgical complexity and patient-related factors such as smoking and poorly controlled diabetes mellitus [78]. Moreover, the incidence of pseudarthrosis following ACDF varies depending on the number of fused levels [76].

Patients undergoing occipitocervical fusion may be at increased risk of nonunion compared to subaxial procedures, particularly in high-risk populations [79].

Numerous studies have investigated predictive factors associated with an increased risk of non-union following ACDF [73,74,75,76,77,78,79,80,81,82]. Among the most consistently identified risk factors are cigarette smoking, diabetes mellitus, osteoporosis [73,80,81,82,83,84], and preoperative radiographic indicators of bone quality and turnover [74,85]. More recently, Lovecchio et al. evaluated cervical Hounsfield Unit (HU) values using computed tomography (CT), demonstrating that HU measurements vary according to vertebral level and anatomical region. Importantly, several studies have reported a correlation between HU values and the biomechanical properties of vertebral bone, as well as clinical outcomes following spinal instrumentation. In particular, lower HU values have been associated with an increased risk of cage subsidence following ACDF procedures [86,87,88].

Aging and comorbidity burden are also associated with progressive reductions in bone mineral density across cervical vertebrae, highlighting the importance of individualized preoperative assessment [86].

Another key factor in predicting pseudarthrosis is the evaluation of osteogenic potential through biochemical markers of bone turnover. These include alkaline phosphatase (ALP), β-carboxyterminal telopeptide of type I collagen (β-CTX), and hormones regulating bone metabolism, such as calcitonin, parathyroid hormone, and vitamin D, as well as serum calcium and phosphorus levels [73,89].

In addition, as discussed earlier, preoperative radiographic parameters, including sagittal alignment and segmental mobility, may influence fusion outcomes following ACDF as they have been shown to do in the lumbar spine [74,90].

The relationship between biochemical markers of bone metabolism, hormonal status, vitamin D levels, and radiographic predictors of fusion remains incompletely understood and requires further investigation.

Univariate analyses have identified reduced bone formation activity—reflected by lower serum levels of calcium, β-CTX, and N-MID-BGP—as a potential risk factor for nonunion. These findings are consistent with previous studies demonstrating that postoperative administration of teriparatide enhances osteoblastic activity and improves fusion rates [73]. Similarly, higher metabolic bone activity has been observed in patients who achieved successful fusion, suggesting that serum calcium levels may represent a sensitive predictor of postoperative outcomes [73]. Future studies with focus on prospective and longitudinal analyses integrating biochemical markers, radiological assessment, and clinical outcomes may better define their predictive value and underlying biological mechanisms.

Osteobiological agents, defined as biologically active materials designed to promote bone regeneration, are increasingly used in clinical practice; however, their overall efficacy remains debated [91]. For example, some studies have reported higher non-union rates associated with specific cellular bone matrices when used in combination with PEEK cages [91].

More recently, pharmacological approaches have been explored. Glucagon-like peptide-1 (GLP-1) receptor agonists have been associated with significantly lower rates of nonunion between 6 months and 2 years postoperatively [92]. These agents are thought to enhance osteoblastic activity while inhibiting osteoclast-mediated bone resorption, thereby improving the biological environment for fusion. However, further investigation is required to clarify their mechanisms of action and clinical applicability.

Adjunctive therapies, such as pulsed electromagnetic field (PEMF) stimulation, have also demonstrated potential benefits in improving fusion rates, clinical outcomes, and patient satisfaction compared with standard postoperative management [93]. However, current evidence does not show significant differences among rehabilitation protocols following anterior versus posterior fusion procedures, indicating the need for further studies to establish optimized postoperative strategies [93].

Preoperative assessment of bone mineral density (BMD) is essential, as it represents a critical determinant of fusion success [94]. Quantitative computed tomography (QCT) and dual-energy X-ray absorptiometry (DXA) are widely used for osteoporosis evaluation [95,96,97,98]. However, both techniques present limitations, including high costs, radiation exposure, and potential measurement inaccuracies [93]. Furthermore, DXA has limited reliability in anatomically complex regions such as the cervical spine and is therefore not routinely used for site-specific assessment [99].

More recently, vertebral bone quality (VBQ) scores derived from MRI have been proposed as a radiation-free alternative for assessing bone quality. Region-specific thresholds have been suggested, such as 2.75 for the cervical spine and 3.22 for the lumbar spine, providing clinically relevant reference values for preoperative risk stratification [100].

Huang et al. introduced the cervical VBQ (C-VBQ) score as a surrogate marker for bone mineral density, demonstrating a significant correlation with conventional BMD measurements [101]. Similar findings were reported by Wang et al. [102].

However, conflicting evidence exists regarding the reliability of VBQ as a predictor of cervical bone quality. For example, Oezel et al. reported that, although VBQ values were consistent across cervical levels, their correlation with QCT-derived measurements was weaker than expected, suggesting limited reliability as a standalone diagnostic tool [99].

Overall, the current literature is characterized by heterogeneous methodologies and non-standardized thresholds to determine fusion, resulting in substantial interobserver variability and limiting clinical applicability.

## 5. Artificial Intelligence Systems

In recent years, artificial intelligence (AI) has attracted growing interest in spine imaging, including the evaluation of patients undergoing anterior cervical discectomy and fusion (ACDF). This interest is mainly related to a well-known limitation of current postoperative assessment: fusion is still judged largely on the basis of radiological interpretation, which may vary among observers and is not supported by universally accepted quantitative criteria [103]. In this setting, AI-based tools could, in principle, help make image analysis more reproducible and less dependent on subjective evaluation.

At present, however, the evidence remains preliminary. Only a small number of studies have addressed AI applications that are directly relevant to postoperative fusion assessment. Much of the available literature concerns related, but indirect, topics, such as vertebral segmentation, bone mineral density estimation, Hounsfield Unit measurement, fracture detection, or general spine image analysis. These applications may be useful in the broader management of spine patients, but they should not be considered equivalent to a validated method for assessing cervical fusion healing.

A potential direct application of AI in this field is the automated analysis of postoperative CT images. In theory, deep learning algorithms could assist in identifying trabecular continuity, radiolucent lines, bone bridging across the operated segment, and the relationship between the cage, graft material, and adjacent vertebral endplates [103,104]. These are precisely the features that radiologists and spine surgeons currently evaluate when judging whether fusion has occurred. The main advantage of an automated approach would be the possibility of reducing interobserver variability and producing more consistent longitudinal follow-up assessments.

Li et al. investigated AI-based vertebral segmentation using dual-energy CT and quantitative CT in a cohort of 120 patients [103]. Their results showed that automated segmentation could reduce measurement variability compared with manual analysis and improve the consistency of bone density evaluation. Although this type of work is relevant, it should be interpreted with caution in the context of ACDF. The study supports the technical value of AI for image segmentation and quantitative assessment, but it does not yet establish an AI-based diagnostic standard for determining whether a cervical fusion is solid.

This distinction is important. Current AI systems mainly quantify imaging features that may be associated with fusion, but they do not directly prove biological healing. Similarly, no validated AI-derived fusion score, objective fusion percentage, or clinically accepted threshold is currently available for routine use after ACDF. For this reason, AI should presently be regarded as a potential support tool rather than as an autonomous diagnostic method. Expert radiological and clinical interpretation remains essential.

A second group of AI applications is indirectly related to fusion outcome. These include automated assessment of bone quality, extraction of HU values from CT scans, estimation of bone mineral density, and vertebral segmentation [103,104]. Such approaches may be clinically useful because poor bone quality, osteoporosis, and reduced vertebral density are recognized risk factors for cage subsidence, pseudarthrosis, and mechanical failure. Therefore, AI may contribute to preoperative risk stratification, especially if integrated with clinical variables such as age, smoking, diabetes, number of fused levels, and bone metabolism status. Nevertheless, these methods assess risk factors rather than fusion itself.

Other AI systems have been developed for broader spinal imaging tasks [104]. Examples include tools for cervical fracture detection, image triage, vertebral labeling, and automated segmentation. These technologies are relevant to spine radiology and may improve workflow efficiency, but their connection with postoperative cervical fusion assessment is indirect. For this reason, they should be clearly separated from studies specifically addressing fusion healing.

Another limitation is the quality and generalizability of the datasets used to train AI models. Spine imaging datasets are often relatively small, heterogeneous, and acquired with different imaging protocols (Table 2) [104]. Labels may also vary depending on the criteria used by individual radiologists or institutions. These factors limit the external validity of AI tools and make multicenter validation essential before clinical implementation.

Overall, AI represents a promising but still immature approach for the assessment of cervical fusion. Its most realistic short-term role is likely to be supportive: improving segmentation, standardizing measurements, assisting longitudinal comparison, and helping quantify imaging features that are currently assessed visually. However, current evidence is not sufficient to support routine AI-based diagnosis of fusion status after ACDF. Future studies should focus specifically on cervical fusion, use standardized radiological criteria, include adequate follow-up, and validate AI outputs against clinically meaningful outcomes.

Beyond studies more closely related to fusion assessment, several AI-based tools have been developed for broader applications in spinal imaging. For instance, CINA-CSpine (Avicenna.AI) is an FDA-approved software intended to support the detection and triage of cervical spine fractures on CT images [109]. Similarly, Nanox.AI Bone provides automated quantitative and qualitative analysis of spinal CT examinations for musculoskeletal evaluation [110]. These systems are relevant because they show how AI is progressively entering spine radiology; however, their role in the specific assessment of postoperative cervical arthrodesis remains indirect. Their main purpose is fracture detection, workflow prioritization, or general bone analysis, rather than evaluation of bone bridging, radiolucent gaps, or graft/cage incorporation after ACDF.

Other platforms, including open-source frameworks for medical image processing and advanced quantitative imaging software, may also support segmentation, three-dimensional reconstruction, and computational analysis of bone structures [111,112]. These tools can be useful from a methodological point of view, particularly for developing reproducible pipelines for image analysis. Nevertheless, their current clinical relevance for cervical fusion assessment remains uncertain, because the quantitative outputs they provide have not yet been linked to standardized fusion criteria or validated against postoperative clinical outcomes.

The same caution applies to the regulatory and methodological aspects of AI implementation. Guidance documents for AI-enabled Software-as-a-Medical Device (SaMD), together with Good Machine Learning Practice principles, emphasize data quality, transparency, monitoring, and human oversight [113,114,115,116]. These requirements are particularly important in the field of spinal fusion, where imaging protocols, follow-up duration, and definitions of fusion still vary considerably across studies. Without standardized labels and clinically meaningful endpoints, even technically accurate AI models may have limited value in routine decision-making.

Overall, AI has clear potential to improve the objectivity and reproducibility of spinal imaging analysis, but its current role in postoperative cervical fusion assessment remains limited. Most available systems address surrogate or indirectly related features, such as bone quality, segmentation, or fracture detection, rather than fusion healing itself. Future work should therefore move from general spine-imaging applications toward dedicated, clinically validated models for ACDF follow-up. Ideally, these models should be trained and tested on multicenter datasets, use standardized radiological definitions of fusion, and be evaluated against longitudinal clinical and surgical outcomes.

## 6. Current Evidence and Clinical Implications in ACDF

### 6.1. Bone Grafts

Among grafting options, iliac crest autograft still represents the reference standard in terms of osteogenic potential and fusion reliability, although donor site morbidity remains a major limitation. Allografts are widely used due to lower surgical morbidity, but available evidence in ACDF remains heterogeneous, particularly in multilevel procedures.

### 6.2. Cages

PEEK cages remain among the most widely adopted implants due to their radiolucency and favorable elastic modulus, whereas titanium cages may provide superior osseointegration at the cost of increased stiffness.

### 6.3. Fusion Assessment

CT currently represents the most reliable imaging modality for postoperative fusion assessment, particularly when evaluating trabecular continuity and extragraft bridging bone. However, no universally accepted radiological criteria currently exist and, in this regard, the use of AI can prove to be a great resource.

### 6.4. Risk Factors

Smoking, osteoporosis, diabetes mellitus, and multilevel fusion procedures appear to be the most consistently reported predictors of pseudarthrosis in ACDF.

## 7. Conclusions

The current state of the art in cervical spine fusion has been widely explored in the literature, yet no main clear directions seem to appear. In this context, the present review critically examined the biomaterials used for bone grafting and intervertebral cages, outlining their key properties, clinical applications, and commercially available solutions, as well as the main limitations associated with postoperative monitoring.

Despite significant advancements in surgical techniques and biomaterials, the evaluation of spinal fusion remains a major unresolved challenge. Imaging-based assessment is still affected by substantial interobserver variability and, more importantly, by the lack of standardized and quantitative criteria, limiting the ability to reliably determine the progression and completion of the fusion process over time.

In this scenario, artificial intelligence (AI)-based systems represent a promising but still evolving solution. Although these technologies have demonstrated the potential to provide objective, reproducible, and quantitative assessments, their current clinical application remains limited by the absence of validated frameworks, standardized outputs, and large-scale prospective validation.

Consequently, while AI may significantly enhance diagnostic accuracy and reduce observer-dependent variability, it cannot yet be considered a fully reliable tool for autonomous clinical decision-making. The debate on this topic is often overrun by the speed of technological development.

Future research should therefore focus on the development of standardized evaluation protocols, the integration of multimodal clinical and imaging data, and the validation of AI-based models in large, multicenter cohorts. Addressing these challenges will be essential to enable the effective translation of AI-driven tools into routine clinical practice and to fully realize their potential in improving the assessment and management of cervical spine fusion.

## Figures and Tables

**Table 1:** Comparative summary of commercial bone grafts and intervertebral cages used in ACDF: fusion rates, follow-up and complications.

Product/Material	Category	Fusion Rate (%)	Follow-Up	Clinical Considerations	Strength of Evidence
Iliac Crest Bone Graft (ICBG) [3,16]	Autograft	85–97%	12–24 mo	Donor-site morbidity, pain; gold standard for osteogenicity	Moderate-High
Cortical/Cancellous Allograft [16,17,18]	Allograft	72–90%	12–24 mo	Higher failure vs. autograft; immunogenicity risk; no donor morbidity	Moderate
Grafton^®^ DBM [28]	Allograft DBM	~70.4%	24 mo	Variable osteoinductive potential; requires structural support (cage)	Low
DBX^®^ DBM Putty [28]	Allograft DBM	~66.7%	24 mo	Lower fusion rate vs. Grafton^®^ in comparative study; particle migration risk	Low
Bio4^®^ (Allograft Cellular Bone Matrix) [27]	ACBM Allograft	Not separately reported *	12–24 mo	Pseudarthrosis reported with some ACBMs + PEEK cages, promising in multilevel ACDF	Low-Moderate
Osteofil^®^ DBM [24,26]	Allograft DBM	*ND*		Significant inter-product variability; limited standalone clinical data in ACDF	Low
Kiel Surgibone^®^ [31]	Xenograft	Comparable to autograft *	12–24 mo	Fusion rates comparable to autograft in multiple ACDF studies; no donor morbidity	Low
CANCELLO-PURE^®^/BIO-GEN^®^ [30]	Xenograft	*ND*		No specific multicenter evidence for spinal fusion. Data is primarily preclinical or from other anatomical sites.	Low
SmartBone^®^ ORTHO [50,52]	Hybrid (bovine/synthetic)	Limited data in ACDF *		Promising biomechanical profile. High-quality controlled clinical studies in cervical fusion lacking.	Low
Hypro-Oss^®^ ORTHO [54]	Hybrid (xenograft/polymer)	*ND*		Limited evidence in cervical spine fusion; no multicenter RCT available	Low
Tutobone^®^ [55,56]	Bovine xenograft (processed)	Lower than autograft; comparable to other substitutes *	12 mo	Sparse evidence in ACDF; overall clinical outcomes comparable; limited ACDF-specific data	Low
Signafuse^®^ (Bioactive glass) [42]	Synthetic—Bioglass	Superior to conventional *	12 mo	Improved fusion outcomes vs. conventional grafts; limited large-scale ACDF RCT data	Low
Apaceram^®^/HAp ceramics [6,39]	Synthetic—Hydroxyapatite	~80–90% (with cage) *	12–24 mo	Low initial mechanical strength; improves with bone ingrowth; brittle	Low-Moderate
PEEK Cage (standalone) [59,60]	Synthetic polymer cage	72–97%	12–24 mo	Radiolucent; elastic modulus near cortical bone; some higher rates vs. Ti-PEEK at 12 mo	Moderate
Titanium Cage [59,60]	Titanium alloy cage	82–95%	12–24 mo	Superior osseointegration; higher stiffness than bone may cause stress shielding	Moderate
Titanium-coated PEEK Cage [60]	Hybrid cage (Ti + PEEK)	Up to 94.1%	24 mo	Combines osseointegration of Ti with radiolucency of PEEK	Moderate
Lattice-structured/Trabecular Cage [61]	3D-printed Ti/PEEK	Comparable to standard; reduced subsidence *	12–24 mo	Mimics trabecular architecture; reduced subsidence risk.	Low-Moderate

* Indicates that no rigorous standalone comparative clinical data (fusion rate with 95% CI) were identified in the reviewed literature for ACDF specifically. Reported values are derived from narrative synthesis or cross-study comparison. ACDF = Anterior Cervical Discectomy and Fusion; ACBM = Allograft Cellular Bone Matrix; DBM = Demineralized Bone Matrix; HAp = Hydroxyapatite; mo = months; ND = insufficient data; PEEK = Polyetheretherketone; RCT = Randomized Controlled Trial; Ti = Titanium.

**Table 2:** Public spine radiology image datasets. Adapted from Kim et al., Journal of Korean Neurosurgical Society, 2024, under CC BY-NC 4.0 [104].

Source	Modality	Region of Interest	Segmentation/Labels	Data Size	Released Date
SPIDER [105]	MR	Lumbar	Segmentation of vertebrae, disc and spinal canal	447 Sagittal series (218 patients)	2023
Mendeley data [106]	MR	Lumbar	Radiology report	48,345 Axial slices (515 patients)	2019
RSNA cervical fracture [107]	CT	Cervical	Label and segmentation of vertebrae and fractures	3112 Patients	2023
BUU spine [108]	XR	Lumbar	Labels of 4 diseases entities	400	2023

## Data Availability

The original contributions presented in this study are included in this article. Further inquiries can be directed to the corresponding authors.

## Abbreviations

The following abbreviations are used in this manuscript:

AI	Artificial Intelligence
ACDF	Anterior cervical discectomy and fusion
CL	Cervical lordosis
CT	Computed tomography
DBM	Demineralized bone matrix
DXA	Dual-energy X-rays absorptiometry
HU	Hounsfield Unit
QCT	Quantitative computed tomography
